# Investigation of neutrophil infiltration in the acute canine atopic dermatitis model

**DOI:** 10.3389/falgy.2025.1716716

**Published:** 2025-12-16

**Authors:** Chie Tamamoto-Mochizuki, Santosh K. Mishra

**Affiliations:** 1 Department of Molecular Biomedical Sciences, North Carolina State University, Raleigh, NC, United States; 2 Department of Clinical Sciences, North Carolina State University, Raleigh, NC, United States; 3 Department of Small Animal Clinical Science, University of Tennessee, Knoxville, TN, United States; 4 Comparative Medicine Institute, North Carolina State University, Raleigh, NC, United States

**Keywords:** dog, canine atopic dermatitis, house dust mite, neutrophil, skin

## Abstract

Atopic dermatitis (AD) is an inflammatory skin condition associated with chronic itch and inflammation in both humans and animals. While this disease depends upon various immune cell types, the precise role and kinetics of neutrophils remain elusive, particularly in relevant large-animal models. Given a recent report suggesting the involvement of neutrophils in a mouse model, we aimed to characterize the temporal presence and infiltration of these cells in a canine model of house dust mite (HDM)-induced AD. AD was induced in canines via HDM exposure, and skin biopsies were analyzed over a time course (0–96 h) using standard H&E staining and specific immunofluorescent (IF) staining for canine neutrophils. Our results showed general cellular infiltration with the H&E method, while IF further confirmed detectable neutrophil immunoreactivity starting between 24 and 96 h post-challenge in atopic skin. Quantitation demonstrated a significant increase in neutrophil infiltration (cells/mm^2^) in atopic skin at 48 h following HDM exposure compared to baseline (*p* = 0.041). Collectively, our data confirms time-dependent infiltration of neutrophils into the skin of the canine AD model following allergen challenge, supporting the hypothesis that this previously overlooked immune cell may play a role in the acute phase of AD pathogenesis and sensitization.

## Introduction

Atopic dermatitis (AD) is a debilitating skin inflammatory condition and a significant health concern, common in both humans and animals (1, 2). Many immune and non-immune cells have been reported to be involved in AD pathogenesis—including Langerhans cells (or other antigen-presenting cells), T cells, eosinophils, basophils, and mast cells (3); however, the precise role of neutrophils has remained elusive. Recent work by Walsh et al. in mice highlighted neutrophil involvement in generating itch during the development of AD (4). Here, we aim to characterize the presence and temporal kinetics of neutrophils in canine AD.

## Materials and methods

Healthy skin samples were obtained from five healthy male dogs. One skin sample was collected from the flank of each dog, except for one dog, from which two samples were collected from different anatomical sites (flank and abdomen), resulting in a total of six healthy skin samples. Atopic dermatitis skin lesions were induced in four laboratory atopic dogs (two males and two females) by epicutaneous application of *Dermatophagoides farinae* HDM slurry, as previously described (IACUC ID no. 17-005-O) (5). Skin samples were collected at baseline (0 h; before HDM application), and at 24 h, 48 h, and 96 h following HDM provocation. Due to IACUC restrictions, a maximum of four skin samples could be collected from each dog; 24 h and 48 h were chosen to capture the early phase of the reaction, while 96 h was selected to represent the resolution phase of the acute flare. Each sample was divided in half; one half was fixed in 10% neutral-buffered formalin for standard H&E staining, and the other half was snap-frozen for neutrophil immunofluorescence (IF) analysis. Neutrophil IF was performed using a mouse anti-canine neutrophil IgG1 monoclonal antibody (1 μg/mL; clone CADO48A, Monoclonal Antibody Center, Washington State University, Pullman, WA) (6–8), followed by detection with a Cy3-conjugated goat anti-mouse IgG secondary antibody (5 μg/mL; A10521, Life Technologies, Carlsbad, CA, USA). A neutrophil-enriched buffy coat smear from canine peripheral blood was used as a positive control, and a mouse IgG1 isotype control antibody (1 μg/mL; MAB002, R&D Systems, Minneapolis, MN) served as a negative control to verify the sensitivity and specificity of the primary antibody. Nuclei were counterstained with 4′,6-diamidino-2-phenylindole (DAPI), and slides were mounted with ProLong Gold antifade mounting medium (P36930, Invitrogen, Waltham, MA) and sealed with coverslips. All slides were examined using a Leica DM5000 B upright fluorescence microscope with a 20× objective (Leica Microsystems, Wetzlar, Germany). Images from three randomly selected fields per sample were captured, and immunolabeled neutrophils were manually counted using the Cell Counter plugin in ImageJ. The area of each field was measured using the Analyze Particles function in ImageJ, and neutrophil density was expressed as cells per mm^2^. The average from the three fields was used as the representative count for each sample.

## Statistical analysis

The Shapiro–Wilk test was used to assess data normality. Statistical comparisons between healthy and AD skin at each time point were performed using the Kruskal–Wallis test, while differences between baseline (0 h) and different time points within the AD group were analyzed using the Friedman test. Both tests were followed by Dunn's *post hoc* multiple comparison test. A *p*-value < 0.05 was considered statistically significant. All statistical analyses were performed using GraphPad Prism version 10.6.1 (GraphPad, Software Inc., La Jolla, CA).

## Results

**Figure 1:**
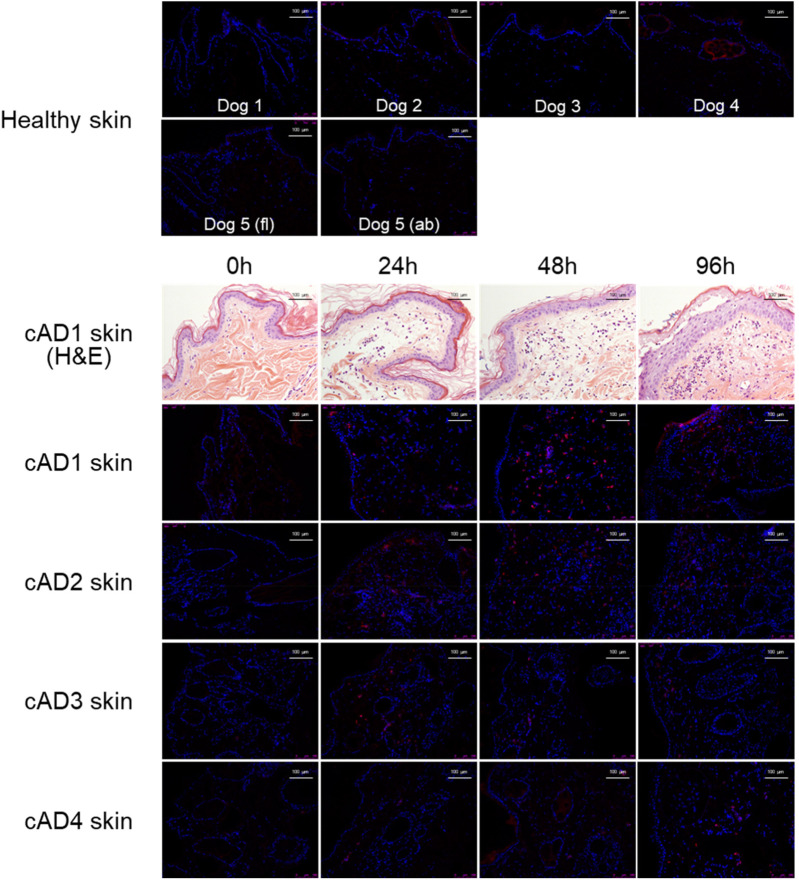
Canine neutrophil immunofluorescent and standard histologic staining (H&E) of healthy and atopic skin of dogs. Immunofluorescent staining targeting canine neutrophils (red) showed no signal in healthy skin or atopic skin prior to house dust mite (HDM) exposure (0 h). Neutrophil immunoreactivity became detectable between 24 and 96 h following HDM exposure. Standard histologic staining (H&E) revealed increased cellular infiltration in the dermis after HDM exposure, although it did not reliably distinguish neutrophils from other cell types. fl, flank skin; ab, abdominal skin; cAD, canine atopic dermatitis; H&E, hematoxylin and eosin staining.

**Figure 2:**
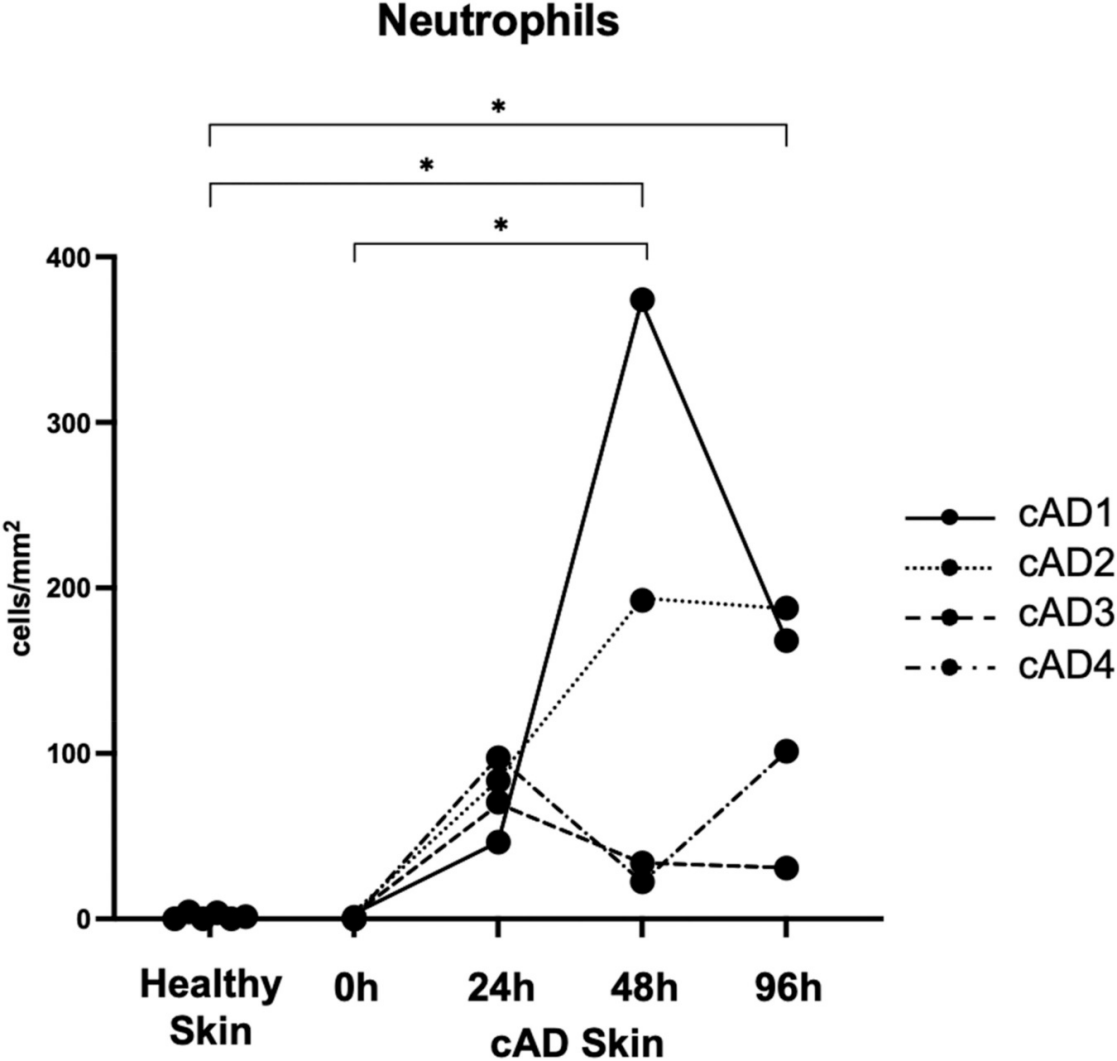
Quantification of neutrophil infiltration in healthy and atopic canine skin. The number of infiltrating neutrophils per area (cells/mm^2^) was quantified. Statistical analysis was performed using the Kruskal–Wallis test (between healthy and AD skin) and the Friedman test (within the AD group) followed by the Dunn *post-hoc* test. A *p*-value < 0.05 (*) was considered statistically significant. A significant increase in neutrophil infiltration was observed in atopic skin at 48 h (48 h) following house dust mite exposure compared to baseline (0 h).

To investigate the presence and infiltration of neutrophils in house dust mite (HDM)-induced AD in canines, we used the standard hematoxylin and eosin (H&E) staining technique to quantify infiltration levels over time following HDM exposure. We observed an increase in general cellular infiltration in the dermis after HDM exposure, which did not consistently differentiate neutrophils from other infiltrating cell types (Figure 1). To confirm neutrophil infiltration, we used immunofluorescent (IF) staining, specifically targeting canine neutrophils (shown in red), which revealed no detectable signal in healthy or atopic skin before HDM exposure (Figure 1, 0 h). Following HDM exposure, neutrophil immunoreactivity became detectable in atopic skin, appearing between 24 and 96 h post-exposure (Figure 1). To quantify the observed infiltration, we determined the number of neutrophils per area (cells/mm^2^) in healthy and atopic canine skin (Figure 2). We found a significant increase in neutrophil infiltration in atopic skin at 48 h following HDM exposure compared to baseline (0 h) (*p* = 0.041; 0 h vs. 24 h, *p* = 0.166; 0 h vs. 96 h, *p* = 0.085; Figure 2). Our quantitative data corroborates the qualitative observations from H&E and immunofluorescent staining, confirming a time-dependent infiltration of neutrophils into atopic skin following allergen challenge. A significant difference between healthy and AD skin was also observed at 48 (*p* = 0.045) and 96 h (*p* = 0.032), but not at 0 (*p* > 0.999) or 24 h (*p* = 0.103; Figure 2).

## Discussion

Neutrophils have traditionally been considered part of a nonspecific innate immune response with limited relevance to AD in both humans and dogs, although the dynamics of other immune cells, such as eosinophils and lymphocytes, have been well characterized in previous studies (9–11). Our data demonstrate the infiltration of neutrophils into the skin of dogs with AD, following the HDM challenge. This infiltration of neutrophils is a time-dependent process, with a gradual increase in neutrophil numbers from 24 to 96 h, while some individual variation was observed within our canine AD colony, as these dogs naturally develop spontaneous AD with differing degrees of disease severity. We used two different methods to evaluate the infiltration of neutrophils qualitatively: the standard staining method, H&E, and specific antibody IF staining for canine neutrophils to identify these cells precisely. The strength of the H&E staining is that it shows general cellular infiltration, but the limitation is that it cannot always reliably distinguish neutrophils from other immune cell types (Figure 1). Although one study demonstrated neutrophil infiltration in the skin lesions of dogs with spontaneous AD, and two studies using the HDM-induced canine AD model showed neutrophil infiltration peaking 6 to 12 h or 24 h after intradermal injection or epicutaneous application of HDM, respectively, all of these studies relied solely on H&E staining for neutrophil identification (9–11). To our knowledge, this is the first study to quantify neutrophil infiltration using neutrophil-specific immunostaining. We used an antibody-based approach on canine skin to confirm and validate the canine-specific neutrophils, which allows accurate detection and quantification. We showed a gradual increase in neutrophil numbers, which reached a significant increase at 48 h post-HDM exposure compared to baseline (Figure 2). Notably, healthy and atopic skin before allergen exposure showed minimal to no neutrophil presence, suggesting that these cells are actively recruited to the site of inflammation, potentially mediated by CD4 tissue-resident memory T cells that may also exist in canine AD skin (12).

Next, we examined the time kinetics of neutrophil infiltration, which arrived later than earlier timepoints and continued to increase at 48–96 h—the final time point of the study. This timing suggests that neutrophil recruitment might represent a secondary wave of inflammation, potentially driven by mediators released during earlier immune responses involving mast cells, eosinophils, or keratinocytes (3). Future research should aim to identify the specific chemoattractant that is responsible for neutrophil recruitment in disease conditions—such as IL-8 (CXCL8) or other CXC chemokines, as well as damage-associated molecular patterns (DAMPs) (13, 14)—that orchestrate this delayed but significant neutrophil infiltration in canine AD. Bacterial colonization is another vital contributor to drive neutrophil responses in humans and animals, and it is common among chronic AD lesions (15).

What is the role of neutrophils in canine AD? Although our data does not directly elucidate any functional or causative role of neutrophils in canine AD, the presence of neutrophils leads to significant functional implications for AD pathogenesis based on previous studies (16). For instance, neutrophils are potent effector cells that might be involved in the release of a wide array of pro-inflammatory mediators, including powerful proteases (such as neutrophil elastases), reactive oxygen species (ROS), and formation of neutrophil extracellular traps (NETs) (17, 18). These substances can directly contribute to tissue damage, amplify inflammation, and potentially exacerbate epidermal barrier dysfunction—a hallmark of AD. Furthermore, neutrophil-derived products may also play a role in AD's cutaneous hypersensitivity and itch characteristics by directly activating sensory neurons or contributing to a neuroinflammatory environment. In a recent study in a mouse model of AD, skin-infiltrating neutrophils were shown to be key initiators of itch and further demonstrated that neutrophil depletion significantly attenuated itch-evoked scratching (4). Overall, the appearance of neutrophils in canine HDM-induced AD models (Figure 2) potentially suggests its interaction with other key immune cells in the skin, such as mast cells, eosinophils, and T cells and non-immune cells such as keratinocytes.

While our study demonstrates neutrophil infiltration in canines, it may have some limitations. The current data focuses only on the presence and quantity of neutrophils but does not fully characterize their causative implication in AD, associated itch, or specific functional contributions *in situ*. This study needs to be followed by future studies employing techniques to assess neutrophil degranulation, NET formation, and the release of specific neutrophil-derived mediators within the skin to investigate their specific contribution to AD pathogenesis. While our findings establish an association between HDM challenge and neutrophil recruitment, causality must be firmly established through *in vivo* neutrophil depletion or inhibition studies in the canine AD model. Such future experiments would clarify their direct role in driving inflammation, pruritus, and barrier compromise in canines.

Another limitation of this study was the skewed sex distribution among healthy control dogs, which were only male. The healthy skin samples were obtained from the local shelter dogs euthanized for population control; therefore, we were unable to control for sex. However, to our knowledge, no studies have reported sex-based differences in immune cell infiltration in canine skin to date.

Lastly, although the focus of this study was to highlight the potential AD-specific role of neutrophils, the cytokine network and immune-cell interactions in AD are far more complex. This network involves multiple immune cell types such as Th2 cells, macrophages, dendritic cells, mast cells, eosinophils, and Th1 cells (19). The recruitment of neutrophils to the AD skin may simply reflect downstream effects of cytokines released by these immune cells. However, neutrophils may also actively contribute to the disease progression, as prior work in a mouse AD model demonstrated that neutrophil infiltration occurred before Th2-cell accumulation (4). Moreover, the depletion of these neutrophils significantly reduced atopic itch, without altering Th2-cells numbers (4), suggesting that neutrophils may exert a more direct role in AD pruritogenesis, independent of Th2-related mechanisms.

Our results highlight the role of neutrophils as significant, late-phase cellular infiltrates in HDM-induced canine AD. The presence of neutrophils at an essential step in their inflammatory development process represents a crucial step forward in generating the complex pathogenesis of canine AD. Future investigation into the specific mechanisms of neutrophil recruitment and their functional consequences could pave the way for identifying novel therapeutic targets to mitigate the chronic inflammation and itch associated with AD in dogs. Given the clinical and immunological similarities between canine and human AD, the canine model provides a valuable platform for exploring the contribution of neutrophils in human AD as well.

## Data Availability

The raw data supporting the conclusions of this article will be made available by the authors, without undue reservation.
